# An unacceptably high burden of anaemia and it’s predictors among young women (15–24 years) in low and middle income countries; set back to SDG progress

**DOI:** 10.1186/s12889-023-16187-5

**Published:** 2023-07-05

**Authors:** Mehari Woldemariam Merid, Dagmawi Chilot, Adugnaw Zeleke Alem, Fantu Mamo Aragaw, Melaku Hunie Asratie, Daniel Gashaneh Belay, Anteneh Ayelign Kibret

**Affiliations:** 1 Department of Epidemiology and Biostatistics, Institute of Public Health, College of Medicine and Health Sciences, University of Gondar, Gondar, Ethiopia; 2 Department of Human Physiology, College of Medicine and Health Sciences, University of Gondar, Gondar, Ethiopia; 3 Department of Women’s and Family Health, School of Midwifery, College of Medicine and Health Sciences, University of Gondar, Gondar, Ethiopia; 4 Department of Human Anatomy, College of Medicine and Health Sciences, University of Gondar, Gondar, Ethiopia

**Keywords:** Anaemia, Risk factors, Young women, Low and Middle Income Countries

## Abstract

**Background:**

Anaemia is a major global public health problem, considerably affects young women in resource limited countries. The available researches on anaemia focused on children, pregnant women, or all women of reproductive age. However, women's biology and life experiences vary dramatically across 15 to 49 years, putting young women bear the higher burden of anaemia, mainly in low and middle income countries (LMICs). Therefore, this study assessed the burden of anaemia among young women (15–24 years) in 24 LMICs which conducted Demographic and Health Surveys (DHS) between 2016 and 2021.

**Method:**

Data analysis was carried out with STATA version 14. The forest plot was used to explore the pooled prevalence of anaemia. Multilevel binary logistic regression was fitted to accommodate the hierarchical nature of the DHS data. Accordingly, a model with lowest deviance (model III) was the best-fitted model. All variables with a *p*-value ≤ 0.2 in the bi-variable analysis were fitted in the multi-level multivariable model. Adjusted odds ratio with 95% CI and *p* < 0.05 were presented to declare statistical significance.

**Result:**

The pooled prevalence of anaemia among young (15–24 years) women in 24 LMICs was 41.58% (95%CI: 34.51, 48.65). Country wise, Mali (62.95%) and Rwanda (14.13%) constitute the highest and lowest prevalence of anaemia. In this study, young women who lived in the poorest wealth status, had no education, were underweight, perceived distance to the health facility a big problem, larger family size, and women who had ever terminated pregnancy were associated with increased odds of anaemia. Whereas, young women who were overweight and not breast feeding had decreased odds of anaemia.

**Conclusion:**

The unacceptably high burden of anaemia among young women setbacks the SDG target; to end all forms of malnutrition by 2030. Therefore, it is highly recommended to take relevant interventions to reduce the burden of anaemia targeted the young women who are uneducated, have low socio-economic status, limited access to health facilities, and lived in larger family size.

**Supplementary Information:**

The online version contains supplementary material available at 10.1186/s12889-023-16187-5.

## Background

Anaemia is a condition in which the number of red blood cells or the haemoglobin concentration within them is lower than normal where their oxygen-carrying capacity is insufficient to meet physiologic needs [1]. A complex interaction among nutrition, infectious diseases, chronic conditions, physiological states, and other factors causes anaemia among young women [2]. It is a major global public health problem that had tremendous impacts on human health, social and economic development [3].

Worldwide, anaemia affects half a billion women of reproductive age and remains a critical challenge mainly in Low and Middle Income Countries (LMCs) [4]. Globally, more than half of young women have suffered from anaemia [5]. It affects approximately one-quarter of young women in developing countries [6]. LMICs contribute to the highest-burden of anaemia, ranging from 13.7% in Ethiopia to 61.5% in Ghana [5].

As to previous scholars, a number of factors are known to affect anaemia among adolescent and young women. Accordingly, age, educational status, marital status, household wealth status, sex of household head, type of toilet facility, source of drinking water, Body Mass Index (BMI), current pregnancy status, currently breast feed, ever had terminated pregnancy, family size, contraceptive use, and media exposure, place of residence, and distance to health facility were key factors that affect anaemia [3, 7–10].

Young women differ from older reproductive-age women in terms of nutritional requirements, duration of menses, and contraceptive use [11–13]. The risk of anaemia increases during adolescent years with the onset of menstruation and pregnancy despite high iron intake demand in response to rapid body growth [14]. Besides, adolescence and young adulthood are the years when young women complete schooling, begin working, and start their families where the impact of anaemia could have considerable maternal and birth adverse outcomes. Hence, anaemia in young women is a serious condition which impedes them from reaching their full potential by reducing educational achievement, labor productivity as well as their cognitive capacity and affect their mental health [15]. Furthermore, in pregnant women, the risk of birth complications and the delivery of low birth weight infants increases with anaemia [9]. Evidences noted that the burden of anaemia among young women is disproportionately concentrated in low socio-economic settings, mostly located in low and middle-income countries, mainly attributable to the complex interplay among the high burden of; infectious diseases, nutritional deficiencies, parasitic infections, and other causes [2, 5, 16]. For instance, gastrointestinal parasites are more common in many developing countries [17].

However, existing researches on anaemia have focused on infants, children, or pregnant women, or in all women of reproductive age (15–49) (WRA). Yet women's biology and life experiences vary dramatically between ages 15 and 49, putting young women at critical group to bear the burden of anaemia. Hence, reducing the burden of anaemia among young women in LMICs by addressing research gap is vital for policy design and the implementation of effective population-level strategies that will help to alleviate the burden of anaemia in low-resource settings. Moreover, this would be helpful to meet the global nutrition targets to reduce anaemia in WRA by 50% by 2025 [18] and to evaluate the progress toward the Sustainable Development Goal (SDG) 2.2 to end all forms of malnutrition by 2030 [19].

Therefore, we assessed the prevalence of anaemia and it’s determinants among young women (15–24 years) from 24 LMICs which conducted Demographic and Health Surveys (DHS) between 2016 and 2021.

## Method

### Study population and data source

This study was based on the most recent dataset from the Demographic and Health Survey (DHS) conducted in 24 low and middle-income countries (LMICs) between 2016 and 2021. The DHS is a nationally representative, cross-sectional survey that provides reliable data on women, men, and children. It uses the same standardized data collection procedures, sampling, questionnaires, and coding, making the results comparable across countries. Therefore, all data were appended together to investigate factors associated with anaemia in women of young age (15–24 years) in LMICs.

**Fig. 1:**
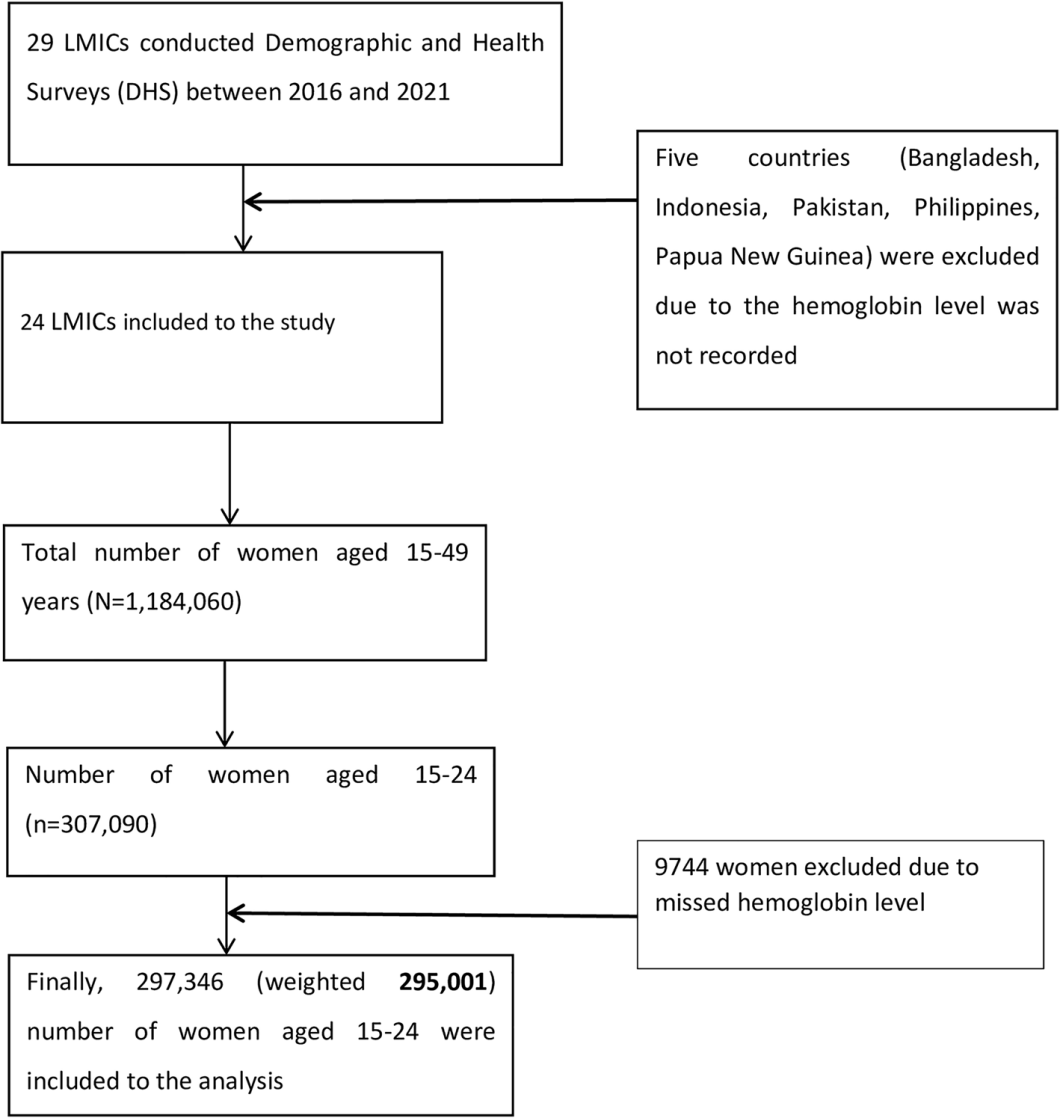
Flowchart showing the inclusion and exclusion criteria of the countries and women included to the study (N=295,001)

The DHS survey employs a two-stage sampling procedure that involves the selection of census enumeration areas from each sampling stratum using a probability proportional to the size of the number of households in each enumeration area in the first stage. In the second stage, households are sampled using systematic random sampling from each enumeration area, which forms the survey clusters. Between 2016 and 2021, 24 LMICs conducted the DHS survey and a total of 307,090 women aged 15–24 years were interviewed. However, this study was limited to young women who had haemoglobin measurement recorded in the data set. As a result, a total weighted sample of 295,001 women of young age (15–24 years) in LMICs was included to the study (Fig. 1).

### Study variables and measurement

#### Outcome variable

Anaemia as measured by the haemoglobin level adjusted for altitude which was recorded in the DHS data was the outcome variable. The haemoglobin level found in the survey data set was already adjusted for altitude using the adjustment formula (adjust =  − 0.032*alt + 0.022*alt2 and adjHb = Hb—adjust (for adjust > 0)). Anaemia among young women (women in the age group of 15–24 years), was measured based on women pregnancy status; for pregnant women a haemoglobin value of < 11 g/dL was considered as and a non-pregnant woman with a haemoglobin value of < 12 g/dL was considered anaemic [3].

For this analysis, women with mild, moderate, and severe anaemia were labelled as anaemic, a haemoglobin levels < 11 g/dl for pregnant and < 12 g/dl for non-pregnant. Hence, anaemia status was dichotomized in to anaemic coded as "1" and not anaemic coded as “0”.

### Independent variables

Based on previous literatures, theoretical and practical significance, both individual and community level variables were included in the study. Accordingly, the individual-level variables considered for our study were; the age of respondent, highest educational level attained, marital status, household wealth status, sex of household head, type of toilet facility, source of drinking water, Body Mass Index (BMI), current pregnancy status, currently breast feed, ever had terminated pregnancy, family size, contraceptive use, and media exposure. On the other hand, place of residence and distance to health facility was the community level variables included in this study.

### Operational definitions

Wealth Index: In the DHS dataset, wealth index was created using principal components analysis coded as “poorest”, “poorer”, “Middle”, “Richer”, and “Richest and taken as it is.

Media exposure: was generated from women's responses to the questions related to the frequency of listening to the radio, watching television, and reading newspapers in a week. It is categorized as "yes" if women had exposure to at least one type of media; radio, newspaper, or television, and "no" otherwise.

Body mass index: was categorized based on the World Health Organization (WHO) cutoff points as underweight (BMI < 18.5 kg/m^2^), normal weight (18.50–24.99 kg/m2), overweight (25.0–29.9 kg/m2), and obese (≥ 30.0 kg/m2).

Distance to health facility: Recorded as a big problem and not a big problem in the dataset was taken without change, which is respondents’ perception during the survey whether they perceived the distance from their home to the nearest health facility to get self-medical help as a big problem or not.

Type of toilet facility: Recoded into two categories as “unimproved and “improved” using the DHS guide.

Source of drinking water: By using the DHS guide it was recoded into two categories as “unimproved” and “improved source”.

### Data processing and analysis

We extracted datasets from 24 LMICs’ PR data files and appended them to generate pooled data. STATA version 14.2 was used to clean, recode and analyse the data. Since the DHS data are hierarchical, i.e., individuals were nested within communities, a multilevel binary logistic regression model was fitted to identify significantly associated factors with anaemia among young women.

Accordingly, four models were constructed comprised of the null model (model 0) without any explanatory variables, Model I with individual independent variables only, Model II with community-level factors only, and Model III with both individual-level and community-level variables. Since the models were nested comparison was made using deviance (− 2 log-likelihood) where model III had lowest deviance and hence was the best. Intra-cluster correlation coefficient (ICC), Median Odds Ratio (MOR), and Proportional Change in Variance (PCV) were applied to measure the degree of heterogeneity and variation between clusters.

Data analysis was carried out with STATA version 14. Descriptive analysis was carried out using frequency and percent distribution of the sample for each of the variables. The forest plot was used to explore the pooled prevalence of anaemia among young women. To identify associated factors of anaemia, we used multilevel binary logistic regression because DHS data are hierarchical, i.e., individuals were nested within communities.

All variables with a *p*-value ≤ 0.2 in the bi-variable analysis were fitted in the multi-level multivariable model. Adjusted OR (AOR) with 95% CI and *p* < 0.05 were presented to declare statistically significant factors for anaemia among young women.

## Result

### Socio-demographic characteristics of the women

A total of weighted 295,001 young (19–24 years) women from 24 low and middle income countries were included in the study. Of these, half (51.13%) were aged 15 to 19 years. More than two-third (68.69%) of the women were rural residents. One-fifth (19.52%) and 17.81% of women were from poorest and richest households, respectively. Only 8.27 percent of the women had no education and close to two-third (62.54%) of them were never in union at the time of the survey. Respectively, 69.83 and 70.77 percent of the women had access to improved water and toilet. One-fifth (21.34%) of the young women had no media exposure. Regarding the women’s BMI status, 28.18% and 10.49% of them were underweight and overweight, respectively (Table 1).

**Table 1:** Socio-demographic characteristics of young women in low and middle income countries, 2022 (295,001)

Variables	Categories	Frequency (%)	Weighted Frequency (%)
**Individual level factors**			
**Age in years**	15–19	152,529 (51.30)	150,822 (51.13)
	20–24	144,817 (48.70)	144,179 (48.87)
**highest educational level**	no education	25,144 (8.46)	24,408 (8.27)
	primary	33,660 (11.32)	33,148 (11.24)
	secondary	194,322 (65.35)	189,939 (64.39)
	higher	44,220 (14.87)	47,506 (16.10)
**Marital status**	never in union	190,996 (64.2)	184,488 (62.54)
	married	102,943 (34.62)	107,223 (36.35)
	divorced/windowed	3,407 ( 1.15)	3,291 (1.12)
**Wealth status**	poorest	64,018 (21.53)	57,577 (19.52)
	poorer	67,956 (22.85)	62,987 (21.35)
	middle	62,647 (21.070	61,775 (20.94)
	richer	55,758 (18.75)	60,110 (20.38)
	richest	46,967 (15.80)	52,551 (17.81)
**Sex of household head**	Male	241,666 (81.27)	239,892 (81.32)
	Female	55,680 (18.73)	55,109 (18.68)
**Type of toilet facility**	Improved	209,553 (70.47)	205,998 (69.83)
	Unimproved	87,79 (29.53)	89,003 (30.17)
**Source of drinking water**	Improved	202,823 (68.21)	208,768 (70.77)
	Unimproved	94,523 (31.79)	86,236 (29.23)
**Family size**	< 5	97,225 (32.70)	97,006 (32.88)
	5–10	182,411 (61.35)	179,209 (60.75)
	> = 10	17,710 (5.96)	18,785 (6.370)
**Had media exposure**	Yes	232,222 (78.10)	232,053 (78.66)
	No	65,122 (21.90)	62,943 (21.34)
**Current pregnancy status**	Yes	19,014 (6.39)	19,457 (6.60)
	No or unsure	278,332 (93.61)	275,544 (93.40)
**Currently breast feeding**	Yes	51,190 (17.22)	52,463 (17.78)
	No	246,156 (82.78)	242,538 (82.22)
**Body Mass Index (BMI)**	underweight	79,428 (27.25)	81,521 (28.19)
	normal	183,679 (63.02)	177,299 (61.32)
	overweight	28,344 (9.73)	30,340 (10.49)
**Contraceptive use**	Yes	43,424 (14.60)	45,472 (15.41)
	No	253,917 (85.40)	249,525 (84.59)
**Ever had terminated pregnancy**	Yes	12,172 (4.09)	13,265 (4.50)
	No	285,174 (95.91)	281,737 (95.50)
**Took iron during pregnancy**	Yes	63,672 (84.66)	65,643 ( 84.77)
	No	11,538 (15.40)	11,790 (15.23)
**Health insurance coverage**	Yes	68,678 (23.74)	63,012 (21.96)
	No	220,567 (76.26)	223,90 (78.04)
**Community level factors**			
**Place of residence**	Urban	77,654 (26.12)	92,364 (31.31)
	Rural	219,692 (73.88)	202,637 (68.69)
**Distance to health facility**	no problem	213,211 (71.70)	218,561 (74.09)
	big problem	84,135 (28.30)	76,440 (25.91)

### Prevalence of anaemia among young women

**Fig. 2:**
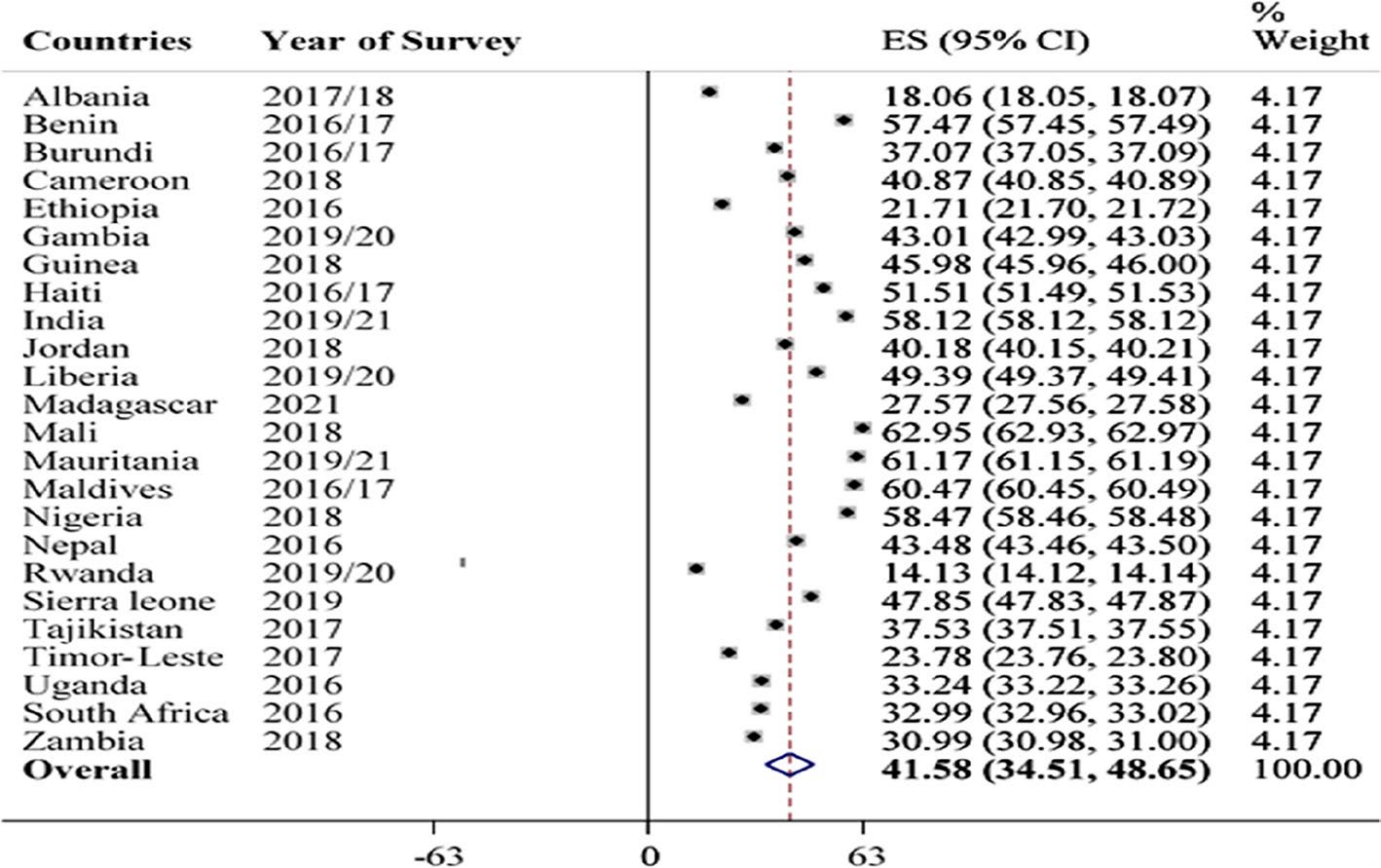
Prevalence of anaemia among young women by countries in low and middle income countries, 2022 (295,001). ***ES****-Prevalence of Anaemia; ****CI****-confidence interval*

In this study, the pooled prevalence of anaemia among young [15–24] years women from 24 low and middle income countries was 41.58% (95%CI: 34.51, 48.65). Among the countries, Mali constitutes the highest (62.95%) burden of anaemia whereas Rwanda was the country with low prevalence (14.13%) of anaemia among young women (Fig. 2).

### Multilevel logistic regression analysis of anaemia among young women in LMICs

The ICC value in the null model was 0.148, indicating that 14.8% of the total variability in anaemia was attributable to between-cluster variability, while about 86.2% was attributable to individual differences. Model III was the best-fitted model because it has the highest log likelihood (-184,170) and the lowest deviance (368,340) value (Supplementary table 1).

Accordingly, we used the final model (the best-fitted model) to assess the determinants of anaemia among young women in LMCs. All variables except sex of the household head, marital status, and contraceptive use were significant in the bi-variable analysis. Age, educational status, wealth index, family size, currently breast feeding, currently pregnant, BMI, ever had terminated pregnancy, distance to health facility were significantly associated with anaemia. As such, the odds of anaemia among young women were 1.46 (AOR = 1.46; 95% CI: 1.41, 1.51) for poorest, 1.32 (AOR = 1.32; 95% CI: 1.28, 1.36) for poorer, 1.23 (AOR = 1.23; 95% CI: 1.20, 1.27) for middle, and 1.14 (AOR = 1.14; 95%CI: 1.11, 1.18) for women from richer household compared to those women from richest household. Similarly, the odds of anaemia were 1.14 (AOR = 1.14; 95% CI: 1.12, 1.16) for underweight, 0.82 (AOR = 0.81; 95% CI: 0.9, 0.84) for overweight women compared to normal weight. The odds of anaemia among women who did not breast feed were decreased by 14% (AOR = 0.86; 95% CI: 0.84, 0.88) compared to their counterparts. The odds of anaemia was (AOR = 1.32; 95% CI: 1.27, 1.37) among women with no education compared to women who attained higher education. Also, the odds of anaemia was 1.05 (AOR = 1.05; 95% CI: 1.03, 1.08) for women who perceive distance to health facility was a big problem compared to their counterparts (Table 2).

**Table 2:** Multivariable multilevel logistic regression analysis of individual-level and community-level factors associated with anaemia among young women in LMCs; 2022 (*N* = 25, 001)

Variables	Categories	**AOR [95% CI]**
**Individual level factors**		
**Age in years**	15–19	**1.06 (1.04,1.09)**
	20–24	Reff
**Educational level**	no education	**1.32 (1.27,1.37)**
	Primary	1.01 (0.97,1.04)
	Secondary	**1.11 (1.09,1.14)**
	Higher	Reff
**Wealth status**	Poorest	**1.46 (1.41,1.51)**
	Poorer	**1.32 (1.28,1.36)**
	Middle	**1.23 (1.20,1.27)**
	Richer	**1.14 (1.11,1.18)**
	Richest	Reff
**Type of toilet facility**	Improved	Reff
	Unimproved	1.01 (0.98,1.03)
**Source of drinking water**	Improved	0.99 (0.9,1.01)
	Unimproved	Reff
**Family size**	< 5	Reff
	5–10	**1.08 (1.06,1.15)**
	> = 10	**1.19 (1.15,1.24)**
**Had media exposure**	Yes	Reff
	No	1.02 (0.99,1.04)
**Currently pregnant**	Yes	Reff
	No or unsure	**1.06 (1.02, 1.09)**
**Currently breast feeding**	Yes	Reff
	No	**0.86 (0.84,0.88)**
**BMI**	Underweight	**1.14 (1.12,1.16)**
	Normal	Reff
	Overweight	**0.81 (0.79,0.84)**
**Ever had terminated pregnancy**	Yes	**1.08 (1.04,1.13)**
	No	Reff
**Health insurance coverage**	Yes	0.91 (0.89,0.93)
	No	Reff
**Community level factors**		
**Place of residence**	Urban	Reff
	Rural	0.98 (0.96,1.01)
**Distance to health facility**	no problem	Reff
	big problem	**1.05 (1.03,1.08)**

*AOR* Adjusted odds ratio*, CI* Confidence interval*, Reff* Referenc

## Discussion

Anaemia is a serious global public health problem, considerably affecting women in resource limited countries. The current study assessed the burden of anaemia and it’s determinants among adolescent and young women in 24 LMICs using DHS survey conducted between 2016 and 2021.

Accordingly, the pooled prevalence of anaemia among young (15–24 years) women from 24 LMICs was 41.58% (95%CI: 34.51, 48.65). By countries, Mali (62.95%) and Rwanda (14.13%) constitute the highest and lowest burden of anaemia, respectively.

The prevalence of anaemia in our study was much higher compared to the global estimate (29.9%) among women of reproductive age in 2019 [20]. Although there has been a gradual decline in the prevalence of anaemia among women of reproductive age (WRA) across the LMICs between 2000 and 2018 [16], our finding noted an increased burden of the condition.

According to WHO, the population is classified into four groups with respect to anaemia: 1) no public health concern (prevalence <  = 4.9%); 2) a mild public health concern (5–19.9%); 3) a moderate public health concern (20.0% to 39.9%); and 4) a severe public health concern (prevalence >  = 40.0%) [21]. Hence, it is clear that anaemia is a critical public health problem and one can predict that it would be far more difficult to reach the GNT target of reducing anaemia in WRA by 50% in 2025 implied that the young women are key subset of the WRA to be addressed. Although countries are expected to reduce the burden of diseases and conditions including anaemia with advancements in socio-economic status and technologies over time, we have noticed that the current estimate of anaemia among young women was higher compared to a report from previous report ranging from 5 to 50% in LMICs [5]. This is a striking alarm that calls countries to take strategic interventions relevant to mitigate the on-going burden of anaemia, particularly on young women where human capital development is consolidated and family formation begins.

In our study, we also found that Mali was the country with highest (62.95%) prevalence of anaemia. This can be explained in that previous studies have reported that socio-cultural food restrictions, parasitic infections such as malaria, hookworm, and shistosomiasis, and some sexually transmitted infections (abnormal vaginal discharge) were responsible for high burden of anaemia in the country [22, 23].

Through this study, we have also identified different individual and community level socio-demographic and related factors which significantly affect the prevalence of anaemia among adolescent and young women in LMICs. Cognizant of this, household wealth status, women’s highest level of education, BMI, breast feeding status, women’s perception on distance to health facility, family size, and weather a woman had ever terminated pregnancy were the factors which significantly affect the prevalence of anaemia among young women in LMICs.

We found that the odds of anaemia among young women with no education were higher compared to those women who attained higher level of education. Similarly, women from the poorest to richer wealth indexes were more likely to have anaemia compared to the richest group of women. These findings are in agreement to previous studies conducted in LMICs including, in South Africa [24], India [25], Ethiopia [7], Nepal [26], Nigeria [27], and Pakistan [28] linking socioeconomic factors with anaemia.

Education has a strong correlation with wealth. The more likely that people get educated, the better chance they secure job and earn more income for completing at least a college degree equates to a better salary [29]. As such, women with better education and financial capacity probably have better knowledge and healthy behaviour which in turn encourages them to adopt healthier lifestyles such as eating nutritious food, better health decision-making, and better hygienic habits [7]. These situations potentially result in better access to nutritious food and better healthcare services that contribute to the prevention and control of anaemia in women with higher education and the richest wealth status compared to women with lower education and wealth status. Therefore, anaemia control programs such as free food supplements and nutrition counselling among women of lower education and marginalized poor women in LMICs will potentially help in reducing the burden of anaemia in the setting.

The other factor associated with anaemia was history of pregnancy termination (abortion), in which women who had history of abortion were more likely to have anaemia than women who do not have it. Aligned with, a study in Trinidad and Tobago, an island country in the Caribbean, found that women with 2–3 terminated pregnancies were more likely to have anaemia than those who didn’t so [30]. The increased probability of women with a history of pregnancy termination having anaemia may be related to post termination bleeding resulting in an increased risk of anaemia [31]. Post- pregnancy termination haemorrhage might have resulted from blood loss due to cervical or vaginal laceration, and uterine atony brought about termination of pregnancy [31].

History of breast feeding was found to be positively associated with anaemia among young women and this was in agreement with findings from other low-income countries [32, 33]. There is evidence that a lactating woman needs to consume more calories with a more diversified diet than non-lactating women [34]. Despite such high calorie demand, studies from Pakistan have reported that women during pregnancy and lactation tend to avoid some food (due to misconceptions and beliefs) such as beef, eggs, fish and citrus fruits as these are considered hot and could have ill effects on their babies [35, 36]. Thus, the high intake demand confounded with avoiding to take some food during pregnancy and lactation could result in increased risk of having anaemia among young women.

Moreover, the amenorrhea associated with full exclusive breastfeeding is generally assumed a protective factor for iron status; insufficient intake of adequate diet could eventually leads to worse iron status and anaemia [37].

Therefore, to address the complication of pregnancy termination such as haemorrhage, assessment of women's hemodynamic status and anticipation of possible blood transfusion are recommended [38].

In agreement with previous studies [39, 40], the odds of having anaemia among young women who perceived distance to a health facility as a big problem were higher than their counterparts. This could be explained in that women living far from a health facility may be linked to poor access to health information and education, diagnosis and treatment, and utilization of health services for diseases and conditions including for anaemia [41, 42].

In our study, a woman who is member of a household with a family size of five or more had a higher likelihood of having anaemia compared to a woman from a household with < 5 family size. This finding was similar to the result of studies in Ethiopia [43] and Pakistan [28] where women with five or more family members had a two-fold increased risk of anaemia. Evidences revealed that as family size increases, there will be an increase in household food insecurity, more communicable diseases, reduced nutrient consumption, often times in extended families [43, 44].

Our study also noted that young women who had lower BMI than normal had higher odds of anaemia whereas overweight women had lower odds of anaemia. This finding was consistent with previous studies in Chines women [45], in Indonesia [46], but contradicts with Colombian women [47]. It is evidenced that women in under nutrition state, will have an increased activity of glycolytic enzymes such as hexokinase, pyruvate kinase and glucose 6-phosphate dehydrogenase (G6PD) which alters membrane permeability, leading to the breakdown of red cells and leading to iron deficiency anaemia [46]. Besides, being underweight may be a reflection poor nutritional status including low iron intake and depletion of micronutrients, collectively results in occurrence of anaemia.

### Strength and limitation of the study

The following are some of the strengths of our study to mention. Firstly, the study was based on a weighted and most recent representative DHS data across the various LMICs. Secondly, the multilevel analysis was employed to accommodate the hierarchical nature of the DHS data and provide a reliable estimate. Moreover, the study has pointed out an insight for policy-makers and program planners to design appropriate intervention strategies LMICs which are relevant to support the global initiatives in an effort to reduce the burden of anaemia in key population segment-the adolescent and young women.

However, our study has some noteworthy limitations to be considered including, the nature of the survey in that the DHS survey was based on respondents’ self-report, which might have the possibility of recall bias. In addition, the temporal relationship between anaemia and the independent variables may not be ascertained. Moreover, some potential confounders such as infections (malaria, intestinal parasites, and some chronic diseases) were not recorded in the DHS and thus were not adjusted in the analysis in this study.

## Conclusion

The unacceptably high prevalence of anaemia among young women in LMICs pose a striking challenge towards the move to reduce anaemia in WRA by 50% by 2025-the GNT target and end all forms of malnutrition by 2030-the SDG. It is therefore an embarking alarm to stakeholders and initiatives to revise current strategies to reduce the burden of anaemia and calls for a special attention to address adolescent and young women- the key population segment in which human capital development is consolidated and family formation begins.

## Supplementary Information

**Additional file 1: Supplementary table 1.** Model comparisons, multivariable multilevel logistic regression analysis of individual-level and community-level factors associated with anaemia among young women in LMCs; 2022 (N=25, 001).

## Data Availability

Data are available online in a public, open-access repository (www.measuredhs.com/data).

## Abbreviations

AOR: Adjusted Odds Ratio
DHSs: Demographic and Health Surveys
GNT: Global Nutrition Target
ICC: Intra-cluster correlation coefficient
MOR: Median odds ratio
PCV: A proportional change in variance
PR: Personal record
SDG: Sustainable Development Goal
WHO: World Health Organization
