# Supplementary material for: An unacceptably high burden of anaemia and it’s predictors among young women (15–24 years) in low and middle income countries; set back to SDG progress
Source: BMC Public Health. 2023 Jul 5;23:1292. doi: 10.1186/s12889-023-16187-5 (PMC10321004; doi:10.1186/s12889-023-16187-5)
Supplement: Supplementary file 1 — Additional file 1: Supplementary table 1. Model comparisons, multivariable multilevel logistic regression analysis of individual-level and community-level factors associated with anaemia among young women in LMCs; 2022 (N=25, 001). [file 12889_2023_16187_MOESM1_ESM.docx]

**Supplementary table 1:** Model comparisons, multivariable multilevel logistic regression analysis of individual-level and community-level factors associated with anaemia among young women in LMCs; 2022 (N=25, 001).

| Variables | Categories | Null model | Model I  AOR [95% CI] | | | | Model II  AOR [95% CI] | | Model III  AOR [95% CI] |
| --- | --- | --- | --- | --- | --- | --- | --- | --- | --- |
| Individual level factors | | |  | | | |  | |  |
| Age in years | 15-19 |  | 1.07 (1.04,1.09) | | | | --------------- | | **1.06 (1.04,1.09)** |
|  | 20-24 |  | Reff | | | | --------------- | | Reff |
| Educational level | no education |  | 1.33 (1.27,1.40) | | | | --------------- | | **1.32 (1.27,1.37)** |
|  | Primary |  | 1.07 (0.97,1.05) | | | | --------------- | | 1.01 (0.97,1.04) |
|  | Secondary |  | 1.12 (1.09,1.14) | | | | --------------- | | **1.11 (1.09,1.14)** |
|  | Higher |  | Reff | | | | --------------- | | Reff |
| Wealth status | Poorest |  | 1.58 (1.46,1.70) | | | | --------------- | | **1.46 (1.41,1.51)** |
|  | Poorer |  | 1.39 (1.30,1.50) | | | | --------------- | | **1.32 (1.28,1.36)** |
|  | Middle |  | 1.25 (1.16,1.34) | | | | --------------- | | **1.23 (1.20,1.27)** |
|  | Richer |  | 1.15 (1.07,1.23) | | | | --------------- | | **1.14 (1.11,1.18)** |
|  | Richest |  | Reff | | | | --------------- | | Reff |
| Type of toilet facility | Improved |  | Reff | | | | --------------- | | Reff |
|  | Unimproved |  | 1.01 (0.98,1.03 | | | | --------------- | | 1.01 (0.98,1.03) |
| Source of drinking water | Improved |  | 0.99 (0.97,1.01) | | | | --------------- | | 0.99 (0.9,1.01) |
|  | Unimproved |  | Reff | | | | --------------- | | Reff |
| Family size | <5 |  | Reff | | | | --------------- | | Reff |
|  | 5-10 |  | 1.08 (1.06,1.11) | | | | --------------- | | **1.08 (1.06,1.15)** |
|  | >=10 |  | 1.19 (1.15,1.24) | | | | --------------- | | **1.19 (1.15,1.24)** |
| Had media exposure | Yes |  | Reff | | | | --------------- | | Reff |
|  | No |  | 1.02 (0.99,1.05) | | | | --------------- | | 1.02 (0.99,1.04) |
| Currently pregnant | Yes |  | Reff | | | | --------------- | | Reff |
|  | No or unsure |  | **1.06 (1.02,1.09)** | | | | --------------- | | **1.06 (1.02, 1.09)** |
| Currently breast feeding | Yes |  | Reff | | | | --------------- | | Reff |
|  | No |  | 0.94 (0.90,0.97) | | | | --------------- | | **0.86 (0.84,0.88)** |
| BMI | Underweight |  | 1.12 (1.09,1.16) | | | | --------------- | | **1.14 (1.12,1.16)** |
|  | Normal |  | Reff | | | | --------------- | | Reff |
|  | Overweight |  | 0.81 (0.79,0.84) | | | | --------------- | | **0.81 (0.79,0.84)** |
| Ever had terminated pregnancy | Yes |  | 1.08 (1.04,1.13) | | | | --------------- | | **1.08 (1.04,1.13)** |
|  | No |  | Reff | | | | --------------- | | Reff |
| Health insurance coverage | Yes |  | 0.91 (0.89,1.93) | | | | --------------- | | 0.91 (0.89,0.93) |
|  | No |  | Reff | | | | --------------- | | Reff |
| Community level factors | | | | | | | | | |
| Place of residence | Urban |  | | ----------- | Reff | | | Reff | |
|  | Rural |  | | ----------- | 1.16 (1.14,1.19) | | | 0.98 (0.96,1.01) | |
| Distance to health facility | no problem |  | | ---------- | Reff | | | Reff | |
|  | big problem |  | | ----------- | 1.11 (1.09,1.13) | | | **1.05 (1.03,1.08)** | |
| Random effect | | | | | | | | | |
|  | Variance | 0.5715 | | 0.5393 | 0.5603 | | | 0.5389 | |
|  | ICC | 0.1480 | | 0.1408 | 0.1455 | | | 0.1407 | |
| Model Comparison | | | | | | | | | |
|  | Log likelihood ratio | -194597 | | -184184.09 | | -184184.99 | | | -184170 |
|  | Deviance | 389,194 | | 368,368.18 | | 369,369.98 | | | 368,340 |
| *ICC = Inter cluster correlation coefficient, AOR=adjusted odds ratio; CI= confidence interval, Reff=reference* | | | | | | | | | |
